# Single‐institution report of setup margins of voluntary deep‐inspiration breath‐hold (DIBH) whole breast radiotherapy implemented with real‐time surface imaging

**DOI:** 10.1002/acm2.12368

**Published:** 2018-06-22

**Authors:** Annie Xiao, Jennie Crosby, Martha Malin, Hyejoo Kang, Maxine Washington, Yasmin Hasan, Steven J. Chmura, Hania A. Al‐Hallaq

**Affiliations:** ^1^ The University of Chicago Pritzker School of Medicine Chicago IL USA; ^2^ Department of Radiation and Cellular Oncology The University of Chicago Chicago IL USA; ^3^ Department of Radiation Oncology Loyola Medicine Maywood IL USA

**Keywords:** breast cancer, deep‐inspiration breath‐hold, setup errors, surface imaging

## Abstract

**Purpose:**

We calculated setup margins for whole breast radiotherapy during voluntary deep‐inspiration breath‐hold (vDIBH) using real‐time surface imaging (SI).

**Methods and Materials:**

Patients (*n* = 58) with a 27‐to‐31 split between right‐ and left‐sided cancers were analyzed. Treatment beams were gated using AlignRT by registering the whole breast region‐of‐interest to the surface generated from the simulation CT scan. AlignRT recorded (three‐dimensional) 3D displacements and the beam‐on‐state every 0.3 s. Means and standard deviations of the displacements during vDIBH for each fraction were used to calculate setup margins. Intra‐DIBH stability and the intrafraction reproducibility were estimated from the medians of the 5th to 95th percentile range of the translations in each breath‐hold and fraction, respectively.

**Results:**

A total of 7269 breath‐holds were detected over 1305 fractions in which a median dose of 200 cGy was delivered. Each fraction was monitored for 5.95 ± 2.44 min. Calculated setup margins were 4.8 mm (A/P), 4.9 mm (S/I), and 6.4 mm (L/R). The intra‐DIBH stability and the intrafraction reproducibility were ≤0.7 mm and ≤2.2 mm, respectively. The isotropic margin according to SI (9.2 mm) was comparable to other institutions’ calculations that relied on x‐ray imaging and/or spirometry for patients with left‐sided cancer (9.8–11.0 mm). Likewise, intra‐DIBH variability and intrafraction reproducibility of breast surface measured with SI agreed with spirometry‐based positioning to within 1.2 and 0.36 mm, respectively.

**Conclusions:**

We demonstrated that intra‐DIBH variability, intrafraction reproducibility, and setup margins are similar to those reported by peer studies who utilized spirometry‐based positioning.

## 1 INTRODUCTION

Adjuvant whole breast radiotherapy (WBRT) following lumpectomy improves local control and in some populations overall survival in the treatment of invasive breast cancer.^1^, ^2^ However, collateral toxicities to the heart and lungs remain a significant challenge.^3^, ^4^ Deep‐inspiration breath‐hold (DIBH) harnesses the advantage of organ motion during the respiratory cycle to minimize overlap of the heart and lungs with the treatment fields.^5^ Compared to free‐breathing, DIBH significantly reduces the dose to the heart for left‐sided treatments^5^, ^6^, ^7^ and the ipsilateral lung for right‐sided treatments.^8^ To minimize irradiation of nearby organs‐at‐risk without sacrificing treatment of the target volume, daily patient setup and breath‐hold reproducibility, which can be monitored through cine portal imaging, spirometry or surface imaging (SI), are essential. Even with spirometric‐DIBH as an alternative to voluntary DIBH (vDIBH), breast surface reproducibility varies 1–3 mm.^9^ Surface imaging has the added benefit of being noninvasive and voluntary.

Image guidance is frequently used to detect large setup errors and refine patient positioning in the setting of radiotherapy.^10^ Modalities such as electronic portal imaging devices, cone‐beam CT (CBCT), and surface imaging have been implemented to verify setup accuracy of DIBH patients.^11^, ^12^, ^13^, ^14^, ^15^, ^16^ However, daily shifts in patient setup are inevitable even with image guidance. To buffer these daily setup errors and ensure adequate dosing to target tissue, the planning treatment volume (PTV) incorporates margins to the clinical target volume (CTV) taking into consideration the systematic and random errors of daily setup.^17^ We refer to PTV margins as setup margins, because these calculations serve as a measure of setup reproducibility in this study rather than as values incorporated clinically into radiotherapy plans.

Unlike radiation‐based image guidance systems that measure displacements in bony anatomy, surface imaging provides real‐time tracking of breast position without dosimetric detriment to the patient. To our knowledge, we are the first to calculate setup margins achieved by vDIBH with real‐time surface imaging of whole breast targets, and compare these values to those achieved in peer studies that use spirometric‐DIBH and/or x ray‐based monitoring. Use of a reference surface from the simulation CT scan throughout the entire treatment course allows us to report on systematic and random errors of the breast surface during vDIBH calculated using real‐time surface imaging data.

## 2 MATERIALS AND METHODS

### 2.A Patient selection and simulation

Data from every consenting patient with an intact breast treated with voluntary DIBH at The University of Chicago Medicine from December 2014 through March 2017 were included in this retrospective study. Patients were simulated in the supine orientation and immobilized in customized upper Alpha Cradle molds (Smithers Medical Systems, Canton, OH) with or without the SaBella Flex breastboard (CDR Systems, Calgary, Alberta, CAN), depending on whether it had been implemented. Molds were designed to encompass the upper body, arms, and hands, which were positioned actively (i.e., by gripping pegs) or passively above the head. Therapists coached the patients over three to five breath‐holds to achieve a consistent maximum inhalation position that could be maintained for 15 s whose amplitudes were monitored using the respiratory motion assessment (RPM, Varian Medical Systems, Palo Alto, CA) device placed at midline just above the xiphoid. Two sequential CT scans were acquired on a Brilliance BigBore scanner (Phillips Healthcare, Andover, MA) with 3‐mm slice thicknesses during free‐breathing (FB) and vDIBH. FB scans were used for dosimetric comparisons and to generate a reference surface for initial treatment positioning. DIBH CT scans were used for treatment planning and generation of a reference surface for breath‐hold guidance. Reference surfaces were automatically contoured in Pinnacle v9.0‐9.6 (Philips Systems, Andover, MA) using a CT density threshold of 0.6 g/cm^3^. The reference surface was exported to AlignRT as a DICOM structure file. AlignRT performs some down‐sampling of the 3D vertices used to define the surface as a triangle mesh surface, whose resolution depends upon the anatomical site chosen within the system and the geometry of the surface.

### 2.B Treatment planning

Patients were treated with 6‐MV or 15‐MV photons on a Varian Trilogy linear accelerator with an OBI console (Varian Medical Systems, Palo Alto, CA). Tangential fields (i.e., 2‐field) were optimized with the field‐in‐field technique to improve dose homogeneity throughout the breast. Anterior and posterior oblique supraclavicular fields (i.e., 3‐field) using a mono‐isocentric technique were added to treat nodal targets when deemed appropriate. Dose calculations with correction for tissue heterogeneities were performed in Pinnacle v9.0‐9.6 with the aim of covering the target, with at least 95% of the prescribed dose while minimizing 20 Gy, 30 Gy, and mean doses to the heart and lungs when compared to FB plans per institutional guidelines.^18^ Breast and nodal targets were contoured per the RTOG Breast Cancer Atlas.

### 2.C Surface imaging

The AlignRT v5.0 three‐camera system (VisionRT, London, UK) was used for initial positioning and during treatment to continuously monitor surface displacements in three translational dimensions (A/P: anterior–posterior; L/R: left–right; and S/I: superior–inferior) and in three rotational dimensions (yaw, roll, and pitch). Displacements are calculated automatically by the software following rigid registration using a proprietary iterative closest point algorithm of a single region‐of‐interest (ROI) delineated by the user on the reference surface. The “Entire” ROI including arms and chin was selected for initial FB positioning while the “Breast” ROI mimicking the projection of the tangential fields was selected for treatment verification at the DIBH position.^19^

**Figure 1:**
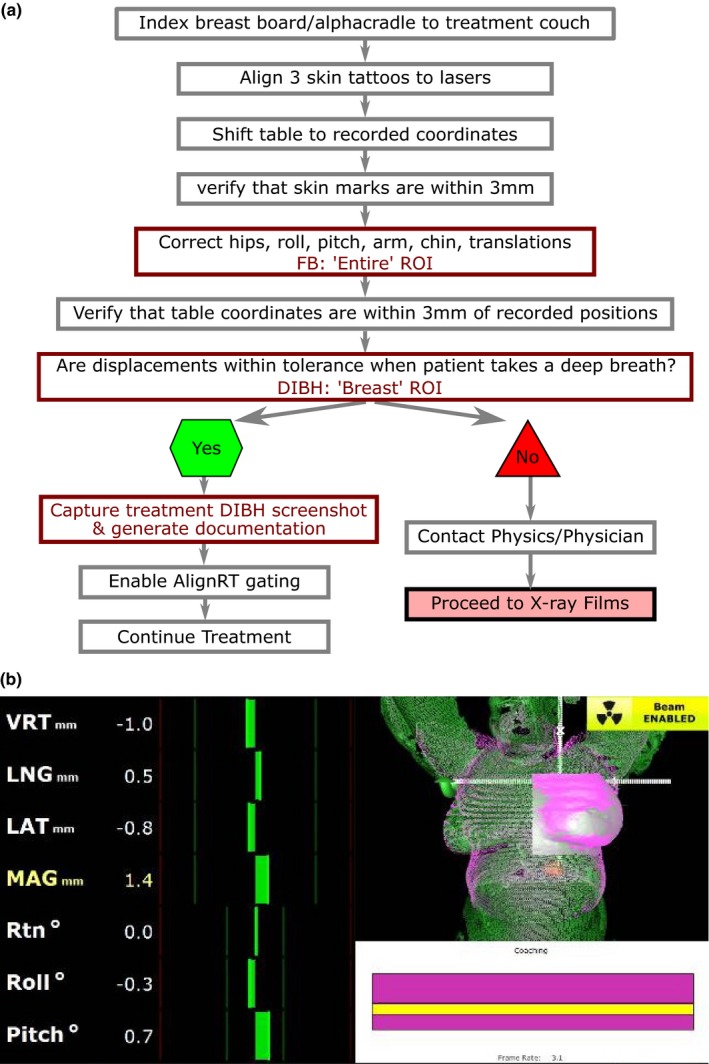
Clinical workflow (a). Therapist's view of DIBH surface in green overlaid on CT‐generated surface in pink (b).

The workflow for patient positioning is depicted in Fig. 1(a). Initial positioning began by aligning skin tattoos to in‐room lasers followed by minimizing the displacements between the real‐time images and the FB reference surface within the “Entire” ROI. Patients were then instructed to hold their breath and coached or adjusted until real‐time displacements of the ‘Breast’ ROI agreed with the DIBH reference surface to within 3 mm/1° in each dimension. Beam gating was enabled during treatment using the tightest 3D thresholds to within a range of 5–7 mm and 2–3°, as determined from the patient's DIBH reproducibility during the first treatment. X‐ray imaging was performed once pretreatment, during the first treatment, and weekly thereafter using an orthogonal kV pair and MV ports, which were used to correct the patient's position as needed. Figure 1(b) illustrates the SI interface used by the therapists, including a typical “Breast” ROI.

**Figure 2:**
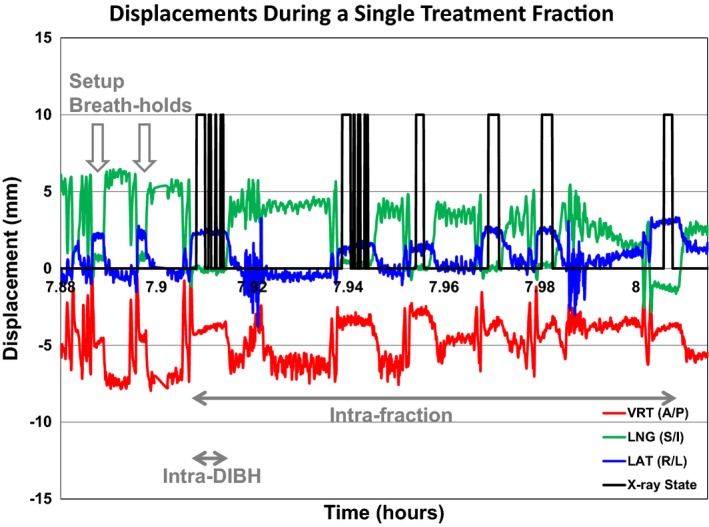
Representative plot of translational displacements in three dimensions (red, green, blue lines) and the beam “ON” state (black line) depicts a single treatment fraction during which there were two setup and six treatment breath‐holds. Intrafraction reproducibility is assessed over all six breath‐holds while intra‐DIBH stability is assessed within each breath‐hold.

Translational and rotational displacements were recorded every 0.3 s and automatically written to an output text file by AlignRT. An in‐house MATLAB (R2016a, The MathWorks, Inc., Natick, MA, USA) script was developed to process these displacements which resulted from registration to the DIBH reference surface. Individual breath‐holds were identified as periods when the radiation beam state was “ON,” which was triggered by the requirement that the patient surface be within tolerance of the reference surface (Fig. 2). Consecutive breath‐holds were distinguished by a temporal separation of at least 5 s, whose value stemmed from knowledge of our clinical workflow.

### 2.D Calculation of setup margins

Translational displacements reported by real‐time surface imaging were used to calculate the means and standard deviations within breath‐holds, per treatment fraction, and over the entire treatment course for each patient. Setup margins in each dimension were calculated by the van Herk equation:

(1) Margin=2.5(Σ)+0.7(σ)

where Σ and *σ* represent the standard deviations of the systematic and random errors, respectively.^17^ Translational displacements are tabulated and the mean and standard deviation for each treatment fraction and across all treatment fractions for each patient were calculated. Systematic errors were computed from the standard deviations of the group mean translational displacements while random errors were computed using the root mean square error of the standard deviations of the translational displacements for each patient.^20^ This formula uses per treatment translations to calculate the setup margin for a population such that 99% of the treatment volume receives 95% of the prescribed dose. To calculate isotropic setup margins, the standard deviations of the systematic and random errors were added in quadrature in each translational dimension. Isotropic margins were used to compare to values in the literature, which were not reported in three independent dimensions. Intra‐DIBH and intrafraction reproducibility (Fig. 2) were computed by taking the median of the 5th–95th percentile range of the translational displacement during a single breath‐hold or during all breath‐holds throughout a single treatment session, respectively, as described by Fassi et al.^9^

### 2.E Statistical analysis

Since setup margins cannot be statistically compared,^21^ the data used to calculate them (i.e., mean errors and standard deviations) were compared among subgroups (right‐sided vs left‐sided and 2‐field vs 3‐field) using a nonparametric one‐way analysis of variance (i.e., Kruskal–Wallis H‐test). To account for increases in the type I error rate (*α *= 0.05) due to multiple testing in each of the three dimensions, the *P*‐value of 0.05 was divided by the number of comparisons made such that results were deemed significant only if *P* < 0.0167 (i.e., *P* < 0.05/3).

## 3 RESULTS

### 3.A Patient characteristics

Table 1 details patient and treatment characteristics. A total of 7269 breath‐holds were analyzed in 1305 fractions across 58 patients, with each patient monitored for an average of 5.95 (±2.44) min per fraction from first to last breath‐hold. Of all treated fractions, 4.9% were not included in the analysis either because: (a) beam gating was not enabled or the beam‐on state was not recorded correctly in the output file (2.5%), or (b) poor reproducibility of the simulated position using the “Breast” ROI necessitated the use of an alternate ROI for real‐time tracking (2.4%). Of the 58 patients, three (5.2%) were resimulated at some point during treatment to improve their daily positioning reproducibility by generating a new CT reference surface from which data was retained for analysis.

**Table 1:** Patient and treatment characteristics

Characteristic	Left breast	Right breast
Number of patients	31	27
Median age (y) [range]	58 [38–85]	51 [31–80]
Body mass index (kg/m^2^)		
Median (range)	26.9	29.1
18.5–25 (normal)	10	6
>25–30 (overweight)	11	8
>30 (obese)	10	13
Stage		
0	6	2
1	10	14
2	13	10
3	2	1
Median dose (Gy)	50	50
Median number of fractions	25	25
No. of fields		
2‐field	17	11
3‐field	14	16
Dosimetric metrics		
Mean heart dose (Gy) in DIBH plan	1.7	–
Mean heart dose (Gy) in FB plan	4.8	–
Ipsilateral lung V20Gy (%) in DIBH plan	21.2%	21.5%
Ipsilateral lung V20Gy (%) in FB plan	26.3%	28.6%
Immobilization device		
Custom only	8	8
CDR Sabella breastboard + custom	23	19
Neoadjuvant chemotherapy		
Yes	13	13
No	18	14
Cardiotoxic chemotherapy		
Herceptin use	5	2
Adriamycin use	9	12
IMN disease	4	9

### 3.B Translational displacements and setup margin calculations

**Figure 3:**
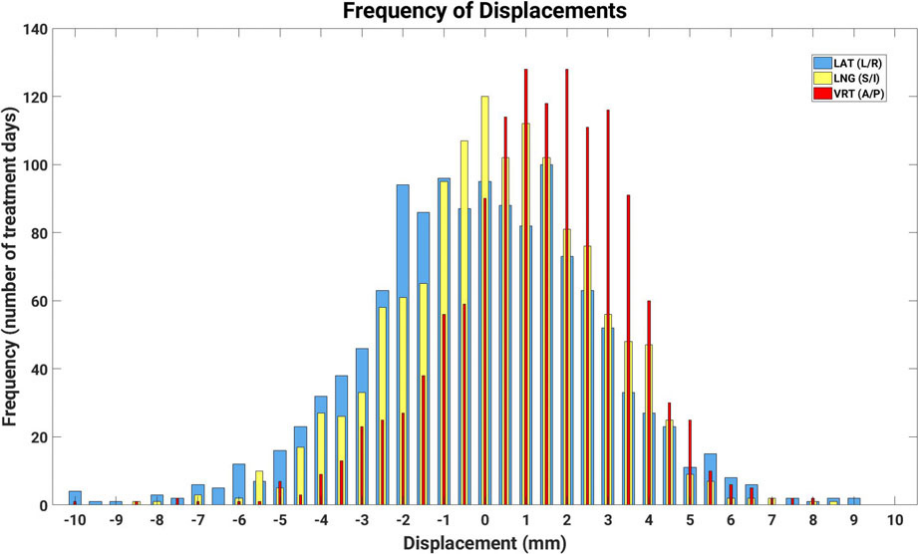
Distribution of translational displacements across all treatment fractions (*n* = 1305). All three dimensions are overlaid using bars of increasing width, with the thinnest for A/P and the widest for R/L.

Figure 3 shows a histogram distribution of mean dimensional displacements for all fractions, which shows a mean translational shift of >1 mm in the A/P direction that does not exist for S/I or R/L. This may be due to a systematic discrepancy between the CT‐generated surface and the true breast surface in the A/P direction.

Table 2 summarizes absolute shifts, setup errors, and calculated setup margins for all patients and subgroups. Percentages of observed shifts within a specified magnitude were also calculated. van Herk's recipe reveals that a uniform 7‐mm margin would encompass greater than 95% of daily shifts, although stricter 5‐mm margins would suffice for A/P and S/I dimensions. In the subgroup analysis, only the mean error in S/I between 2‐field and 3‐field treatments differed (*P* < 0.0167) indicating that systematic S/I errors were significantly smaller for 3‐field treatments. There were no statistically significant differences in body mass index (BMI) among patients in either subgroup.

**Table 2:** Absolute displacements, total, systematic, and random errors, and setup margins for each dimension

	A/P	S/I	L/R
Absolute shifts ±SD, mm	1.98 ±1.45	1.88 ±1.45	2.19 ±1.76
% shifts within 5 mm	96.9	97.1	92.9
% shifts within 6 mm	98.7	98.9	96.4
% shifts within 7 mm	99.3	99.4	98.2
Total setup error (mm)	1.12	0.09	−0.43
Left breast	1.14	0.27	−0.55
Right breast	1.10	−0.11	−0.30
2‐Field	1.41	0.62^a^	−0.56
3‐Field	0.86	−0.40^a^	−0.32
SD of systematic error (mm)	1.41	1.40	1.93
Left breast	1.48	1.34	1.85
Right breast	1.36	1.47	2.04
2‐Field	1.24	1.31	1.83
3‐Field	1.52	1.32	2.04
SD of random error (mm)	1.82	2.00	2.18
Left breast	1.71	1.95	2.16
Right breast	1.93	2.06	2.21
2‐Field	1.70	1.82	2.01
3‐Field	1.92	2.16	2.33
Setup margin (mm)	4.80	4.90	6.35
Left breast	4.89	4.70	6.15
Right breast	4.74	5.12	6.64
2‐Field	4.29	4.55	5.99
3‐Field	5.15	4.81	6.72

a Statistically significant difference using H‐test (*P* < 0.0167).

Comparisons of isotropic setup margins and standard deviations of the systematic and random errors are shown in Table 3 between the left‐sided breast group in this study and those in Conroy et al. 14 and Yang et al. 15, which both use x‐ray monitoring of left‐sided DIBH. The use of surface imaging in this study achieved a comparable setup margin to those in aforementioned peer works.

**Table 3:** Isotropic setup margins and errors for left breast patients comparing SI to x ray positioning using both voluntary and spirometric‐DIBH

	This analysis	Conroy et al.^14^	Yang et al.^15^
DIBH Method	Voluntary	Voluntary	Spirometry
Imaging modality	SI	X ray	X ray
Number of patients	31	30	28
SD of systematic error (mm)	2.7	3.2	3.8
SD of random error (mm)	3.4	2.6	2.1
Setup margin (mm)	9.2	9.8	11.0

### 3.C Intra‐DIBH and intrafraction analysis

Table 4 directly compares the median intra‐DIBH stabilities and intrafraction surface reproducibility assessed by surface imaging on vDIBH in our study to those assessed by infrared markers on spirometric‐DIBH in Fassi et al.^9^ as well as the percentages of each quantity that lie within a specified magnitude. These data report superior intra‐DIBH stability with voluntary DIBH over spirometry‐controlled DIBH, but comparable intrafraction reproducibility between the two DIBH techniques. The data demonstrate that patients can reproduce their breast surface to better than 3 mm in a single breath‐hold. SI thresholds of at least 5 mm are indicated in order to capture the variability in voluntary breath‐holds over an entire treatment fraction (i.e., intrafraction) for 95% of treatments when referenced to the CT‐acquired surface.

**Table 4:** Intra‐DIBH stability and intrafraction reproducibility from 31 left‐sided patients in this analysis compared to those of spirometry‐based results from seven patients as reported on in Fassi et al

	Intra‐DIBH variability (mm)			Intrafraction reproducibility (mm)		
	A/P	S/I	R/L	A/P	S/I	R/L
Fassi et al. (*n* = 7) Surface monitoring + spirometry	1.37	1.78	0.74	2.16	2.30	1.88
This analysis (*n* = 31) SI + voluntary DIBH	0.66	0.58	0.49	2.17	1.98	1.52
% within 2 mm	72.1	76.3	84.6	–	–	–
% within 3 mm	98.2	98.8	99.6	74.6	76.0	88.4
% within 4 mm	99.4	99.6	100.0	88.1	89.5	95.7
% within 5 mm	–	–	–	94.9	96.5	98.2
% within 7 mm	–	–	–	99.2	99.5	99.6

## 4 DISCUSSION

The DIBH techniques offer proven advantages in breast radiotherapy via dosimetric sparing of organs‐at‐risk.^5^, ^6^, ^8^ At our institution, DIBH is currently offered to every supine breast cancer patient treated curatively using surface imaging for real‐time tracking of the “Breast” region‐of‐interest throughout treatment [Fig. 1(b)]. Because our implementation utilizes the CT‐simulated surface as the reference, we are able to quantify both random and systematic errors in real‐time during the beam‐on time, which result from registration of the entire breast surface. When analyzing 1305 fractions from 58 patients, we calculated a setup margin of 4.8 mm (A/P), 4.9 mm (S/I), and 6.4 mm (L/R). Since setup margins are not routinely used for developing breast radiotherapy plans, there is limited data in the literature against which to compare. Alderliesten et al.^13^ compared vDIBH setup errors quantified by SI to daily CBCT for left‐sided breast cancer patients; combining these errors using Eq. (1) yields setup margins of 4.6 mm (A/P), 4.8 mm (S/I), and 5.2 (L/R), which are comparable to our results in A/P (4.9 mm) and S/I (4.7 mm) but not in L/R (6.2 mm). This discrepancy could be because a single‐camera AlignRT system was used in the Alderliesten study resulting in truncation of the “Breast” surface, or due to differences in patient habitus between the two study populations as two‐thirds of patients in this study were overweight or obese (Table 1). Isotropic setup margins were also calculated (Table 4) using Eq. (1) from systematic and random errors reported for left‐sided breast DIBH treatments in two studies.^14^, ^15^ Setup margins in our subset of left‐sided breast cancer patients treated using SI‐monitored vDIBH were comparable to those from Conroy et al., which used x‐ray monitoring of vDIBH. Compared to x‐ray monitoring of spirometric‐DIBH,^15^ vDIBH yielded a 1.8‐mm narrower setup margin as calculated in our study, indicating that spirometric control does not guarantee greater surface reproducibility during breath‐hold. This difference could also be attributed to the fact that radiation‐based imaging measures displacements of underlying bony anatomy rather than of skin, and may not be representative of true breast position.^19^ As shown by others,^14^, ^22^, ^23^ we found excellent patient compliance for voluntary DIBH and did not detect any differences in setup reproducibility for left‐ vs right‐sided breast cancer patients, nor worse reproducibility for 3‐field vs 2‐field treatments. In fact, we observed significant reductions in the longitudinal setup errors for 3‐field treatments (Table 2). This finding was corroborated by Kügele et al.^24^, who postulated that this was related to less tissue deformation around the isocenter location for 3‐field treatments compared to 2‐field treatments, whose isocenters were located in deformable breast tissue. In our study, all patients were able to perform vDIBH and only ~5% required resimulation due to either anatomical or breath‐hold reproducibility changes.

While it might be assumed that spirometric control of DIBH results in exact breath‐hold reproducibility over vDIBH, surface tracking of external infrared thoraco‐abdominal markers on seven patients during spirometric‐DIBH showed intrafraction variation in the breast surfaces on the order of 1.75–2.5 mm with lung volumes.^9^ When comparing intra‐DIBH and intrafraction variability in the breast surface achieved by vDIBH to Fassi et al.^9^, we found slightly better intra‐DIBH stability in vDIBH over spirometric‐DIBH, and comparable intrafraction reproducibility regardless of breath‐hold technique indicating that vDIBH measures up to spirometry. The implication of this finding is tremendous given that vDIBH is far less invasive, and is vastly preferred to spirometry by both patients and therapists.^14^, ^22^

While comparison with Fassi et al.^9^ invites the possibility that vDIBH may offer an advantage with respect to intra‐DIBH stability, Table 4 showed that a 5‐mm threshold is still necessary to account for 95% of intrafraction variability in each dimension, which corresponds to a 7‐mm threshold on the 3D magnitude vector as required by the AlignRT v5.0 software. This is a larger threshold than the 3‐mm^12^ and 5‐mm thresholds^11^, ^25^ reported by others. Potential explanations for these differences are that some studies assessed fewer patients^25^ and/or included chestwall patients whose surfaces are less deformable than intact breast tissue.^12^, ^25^ Another explanation is that certain workflows allowed the reference surface to be re‐acquired during treatment. Gierga et al. reported that 22% of voluntary breath‐holds were out of the 5‐mm tolerance by surface imaging and thus required acquisition of a new reference surface.^26^ Our institutional workflow utilizes the CT‐simulated surface throughout treatment enabling a quantification of both systematic and random errors.

Amidst numerous DIBH implementation and monitoring options, the relative best approach for optimal patient setup and DIBH reproducibility remains uncertain. Surface imaging, however, is a clear frontrunner given that it offers nonradiation‐based, real‐time monitoring both pre‐ and mid‐treatment. Not only does it allow visualization of the entire treatment surface including the chin and arms during initial positioning,^19^ but it enables direct tracking of the treated breast without any surrogate such as bony landmarks used during x‐ray imaging.^11^, ^12^, ^25^ Even when MV cine imaging is used to implement vDIBH, Tang et al.^12^ demonstrated that the correlation between chestwall position on digitally reconstructed radiographs and MV portal images was not perfect (*r* = 0.8) and postulated that this could be due to differences in image resolution between the two modalities. Another advantage of surface imaging is that, like RPM, it can disable the treatment if the patient's position exceeds tolerance limits. In contrast to RPM, which only tracks a single point on the patient's abdomen, SI tracks the entire treated surface in real time. Rong et al.^25^ showed that in contrast to SI, there is a lack of correlation between the chestwall excursion, which they used as a surrogate for the target, and the RPM system. Finally, SI monitors the entire breast target which is deformable. Changes in either positioning reproducibility or breast volume (i.e., due to lymphedema or seroma cavity shrinkage^27^) manifest as translational and/or rotational discrepancies in SI, thereby alerting the treatment team once tolerances are exceeded.^19^ However, the extent of tissue deformation cannot be quantified accurately because of the rigid registration algorithm implemented in AlignRT. Overall, SI serves as an excellent quality monitoring tool. Potential future quality improvement tools from which breast cancer patients could benefit include collision prediction modeling^28^ and facial recognition.^29^ Due to the prevalence of this technology on many treatment units, its high compliance rate demonstrated in our study, and future potential expansions, SI should be considered for implementation of vDIBH.

One limitation of this study is that the reference surface was generated from CT scan data, which may not perfectly match the surface rendered by the SI cameras. A histogram of the distributions of translational displacements (Fig. 3) shows that the distribution of A/P displacements is offset by just over 1 mm, whereas the distribution of S/I and L/R displacements are roughly centered on zero. The shift in distribution of A/P displacements, which has been corroborated by others,^13^ may stem from a systematic bias resulting from the autosegmentation of the CT external surface using a specific CT density. Li et al. observed a larger bias in the A/P dimension than in the other dimensions, which could be as large as 0.8 mm depending on the CT density used to generate the reference surface.^30^ If this were true, the translational shifts reported on in this study would represent an upper limit and in fact, setup reproducibility could be better than the estimates we provide here.

Another limitation is that daily discrepancies from the CT‐simulated position may result in dosimetric deviations from the intended treatment plan. Table 1 demonstrates that vDIBH reduced mean heart dose by 3.1 Gy on average and ipsilateral lung V20 by 5%–7%, which is comparable to other studies.^6^, ^8^, ^12^, ^22^ For patients who do not consistently reproduce their simulated vDIBH position, these dosimetric advantages may not be fully realized. Topolnjak et al. used CBCT to study vDIBH reproducibility and found that although patients do not achieve the heart metrics in the treatment plan, their breath‐holds ensure a consistent dosimetric advantage over free‐breathing.^31^ Others have reported on this sustained advantage of vDIBH compared to free‐breathing to reduce heart dose in this otherwise healthy patient population.^22^, ^32^

## 5 CONCLUSIONS

In this study, we report on our institution's setup margins and DIBH stability and reproducibility achieved by real‐time surface imaging of vDIBH, and compare these values with those in peer studies that use spirometry and/or other image guidance modalities. This study shows that our institution's approach of vDIBH implemented with real‐time surface imaging provides setup reproducibility on par with spirometry, and further recommends the use of a 3D magnitude threshold of 7‐mm based on observed patient reproducibility of breath‐hold. Our findings support the use of voluntary DIBH implemented with real‐time surface imaging using a CT‐generated reference surface as a low barrier, high‐quality option to implementing DIBH protocol and improving dosimetric sparing of organs‐at‐risk in breast radiotherapy.

## CONFLICT OF INTEREST

No conflicts exist.
